# The effects of creatine monohydrate supplementation on creatine transporter activity and creatine metabolism in resistance trained males

**DOI:** 10.1186/1550-2783-12-S1-P43

**Published:** 2015-09-21

**Authors:** Tom Andre, Sarah McKinley-Barnard, Josh Gann, Darryn Willoughby

**Affiliations:** 1 Exercise and Biochemical Nutrition Lab, Department of HHPR, Baylor University, Waco, TX 76798, USA

## Background

Oral creatine supplementation is known to provide numerous benefits, including increases in lean muscle mass, muscular strength, and enhanced performance in various athletic capacities. The creatine transporter is a transmembrane protein that mediates the entry of creatine from the circulation into the muscle cell. Little is understood about the importance of the creatine transporter in controlling the uptake and regulation of creatine within human skeletal muscle. The purpose of this study was to conduct a preliminary examination of the effects of a typical creatine monohydrate supplementation regimen on the activity of the creatine transporter at the transcriptional and translational levels in resistance-trained males.

## Methods

In a double blind manner, nineteen (creatine = 9, placebo = 10) resistance-trained (i.e. thrice weekly, > 1 year prior) men between the ages of 18-30 were randomly assigned by age and body weight to orally ingest packets containing a powdered dextrose placebo (AST Sports Science; Golden, CO) or micronized creatine monohydrate (AST Sports Science; Golden, CO). After baseline strength and body composition testing procedures, participants ingested creatine or placebo at a dose of 0.3 g/kg lean body mass/day (≈ 17-20 g/day) for one week in the loading phase and, immediately post loading phase, a dose of 0.075 g/kg lean body mass/day (≈ 5-7 g/day) during the four week maintenance phase. A four week wash out phase followed the supplementation protocol. The participants followed a periodized 4-day per week resistance-training program split into two upper body and two lower body workouts per week, for a total of nine weeks. A total of five muscle samples were collected: Day 1, 8, 22, 36, and 64; six blood samples were obtained: Day 1, 4, 8, 22, 36, and 64; and nine 24-hour urine samples: Day 1, 4, 8, 15, 22, 29, 36, 50 and 64. Statistical analyses were performed utilizing separate two-way ANOVA for each criterion variable employing a probability level of ≤ 0.05.

## Results

Creatine supplementation induced significant increments in total body mass (p = 0.03) and lean body mass (p = 0.01). A moderate effect size (d = 0.51) was found for strength increase. Significant group × time interactions were found for the elevated levels of urinary creatine (p = 0.01), serum creatine (p = 0.003), and muscle total creatine (p = 0.043) in the creatine group compared to placebo. However, no statistical difference was observed for creatine transporter mRNA (p = 0.78) or protein content (p = 0.36).

## Conclusion

Despite detectable differences in levels of urinary, serum, and muscle total creatine content, a standard creatine supplementation protocol had no apparent effect on creatine transporter mRNA or protein expression following a loading, maintenance, and washout phase. Further investigation is warranted to fully elucidate the regulation of creatine transporter activity.

